# Morphology engineering for novel antibiotics: Effect of glass microparticles and soy lecithin on rebeccamycin production and cellular morphology of filamentous actinomycete *Lentzea aerocolonigenes*

**DOI:** 10.3389/fbioe.2023.1171055

**Published:** 2023-04-06

**Authors:** Anna Dinius, Kathrin Schrinner, Marcel Schrader, Zuzanna Justyna Kozanecka, Henry Brauns, Leon Klose, Hannah Weiß, Arno Kwade, Rainer Krull

**Affiliations:** ^1^ Institute of Biochemical Engineering, Technische Universität Braunschweig, Braunschweig, Germany; ^2^ Center of Pharmaceutical Engineering, Technische Universität Braunschweig, Braunschweig, Germany; ^3^ Institute for Particle Technology, Technische Universität Braunschweig, Braunschweig, Germany

**Keywords:** microparticle-enhanced cultivation, rebeccamycin, *Lentzea aerocolonigenes*, glass particles, morphology engineering, soy lecithin, oxygen profiling

## Abstract

*Lentzea* *aerocolonigenes*, as an actinomycete, is a natural producer of the antibiotic and antitumoral drug rebeccamycin. Due to the filamentous cellular morphology handling in cultivations is challenging; therefore, morphology engineering techniques are mandatory to enhance productivity. One promising approach described in the literature is the addition of mineral particles in the micrometer range to precisely adjust cellular morphology and the corresponding product synthesis (microparticle-enhanced cultivation, MPEC). Glass microparticles are introduced in this study as a novel supplementation type for bioprocess intensification in filamentous organisms. Several investigations were conducted to screen for an optimal particle setup, including particle size and concentration regarding their impact and effects on enhanced productivity, microparticle incorporation behavior into the biopellets, the viability of pellets, and morphological changes. Glass microparticles (10 g·L^−1^) with a median diameter of 7.9 µm, for instance, induced an up to fourfold increase in product synthesis accompanied by overall enhanced viability of biomass. Furthermore, structural elucidations showed that biopellets isolated from MPEC tend to have lower hyphal density than unsupplemented control pellets. In this context, oxygen microprofiling was conducted to better understand how internal structural changes interwind with oxygen supply into the pellets. Here, the resulting oxygen profiles are of a contradictive trend of steeper oxygen consumption with increasing glass microparticle supplementation. Eventually, MPEC was combined with another promising cultivation strategy, the supplementation of soy lecithin (7.5 g·L^−1^), to further increase the cultivation performance. A combination of both techniques in an optimized setup resulted in a rebeccamycin concentration of 213 mg·L^−1^ after 10 days of cultivation, the highest value published so far for microparticle-supplemented shake flask cultivations of *L. aerocolonigenes*.

## 1 Introduction

One major global crisis in our developed world is the fight against antimicrobial resistance due to excessive and irresponsible usage of antibiotics inter alia in the health sector and the livestock and food industries. As the efforts to develop or discover new antibiotics have steadily decreased over the past decades, the situation has become more serious, and bacterial infections have become a genuine threat once again (Ventola, 2015; Sharma et al., 2016; Aslam et al., 2018). Thus, it is imperative to return and actively seek novel antibiotics to avoid running out of scopes of action.

A wide source of numerous known antibiotics is found in actinomycetes, especially filamentous growing genera, such as *Streptomyces* strains; these are natural producers (Genilloud, 2017; Mast and Stegmann, 2019), making them attractive and worthwhile for applied research and industrial applications. However, a special feature of filamentous microorganisms is their growth in complex cellular morphologies ranging from dispersed mycelium to scattered mycelial clumps and dense spherical pellets. To attain the preferred morphology for enhanced product synthesis, various strategies have been developed in the past to address specific modifications (e.g., alteration of pellet size) regarding filamentous cellular morphology (Driouch et al., 2012), generally termed *morphology engineering* (Böl et al., 2021). Although classical methods pay attention to cultivation control on the process level (e.g., alteration of pH value or stirrer configurations) (Krull et al., 2013), the latest strategies focus on the supplementation of additives or substances to the cultivation (Böl et al., 2021; Dinius et al., 2023) to increase the product formation.

Microparticle-enhanced cultivation (MPEC) is one of these techniques, which was first introduced by Kaup et al. (2008). It includes the addition of microparticles of varying types and shapes to cultivation broths of filamentous fungi and bacteria to influence cellular morphology and, thus, enhance productivity and the overall cultivation process (Kaup et al., 2008). Thereby, the positive effect of microparticles was shown, exemplified by the cultivation performance of fungal *Caldariomyces fumago* for chloroperoxidase production. Additionally, other filamentous fungi and one filamentous bacterium were also proven to be positively affected in the same study (Kaup et al., 2008). As of this date, multiple successful examples of MPEC have been published for filamentous fungi and bacteria, as summarized by Laible et al. (2021). Generally, MPEC is associated with a multi-layered effect on mycelial biomass. Substantial changes can be recorded due to MPEC. First, a tight intertwining of microparticles and hyphae was reported in eukaryotic or prokaryotic microorganisms (Walisko et al., 2017; Kuhl et al., 2020). Even total incorporation within the mycelial pellet structure was observed, forming particle cores in the pellet centers (Driouch et al., 2012). As a result, the mycelial structure was proposed to be of lower density due to microparticle incorporation; in combination with the microparticle accumulation in the core, it resulted in an enhanced mass transfer of, e.g., oxygen, through the active pellet layer (Driouch et al., 2012; Kuhl et al., 2020). Second, MPEC reduces the overall pellet size (Driouch et al., 2012; Walisko et al., 2015; Kuhl et al., 2020), which is, in combination with a looser mycelial structure, beneficial for enhanced nutrient and oxygen supply throughout the pellet (Gonciarz et al., 2016). Thus, the oxygen-limited area in the pellet is smaller, resulting in a higher specific surface area and enhanced specific productivity (Driouch et al., 2012). So far, MPEC was not limited to shaking flask applications, but is also found to be advantageous for filamentous cultivation in small and large lab-scale stirred tank bioreactors (30 L) (Kowalska et al., 2020; Gürler et al., 2021).

In this study, the MPEC technique was transferred to application in *Lentzea aerocolonigenes* cultivations. Hereby, the novelty is the introduction of silica (SiO_2_-)-based (soda-lime glass) microparticles (median particle size x_50_ = 7.9 µm) as a reliable particle system with defined characteristics for MPEC to enhance filamentous production efficiency. So far, the most commonly used materials for MPEC are talc (Mg_3_ [(OH)_2_|Si_4_O_10_]), aluminum oxide (Al_2_O_3_), and, more scarcely, titanate (TiO_2_). However, usually little information is provided regarding these materials beyond particle size, although the properties of these metal oxide species differ fundamentally regarding morphological characteristics and physiochemical properties (Laible et al., 2021). Thus, a microparticle system with more consistent particle properties is demanded to better control and modulate the challenging filamentous cultures. Therefore, glass microparticles hold the potential to help better understand the pure physical importance of microparticles beyond their fluctuations regarding morphological aspects such as shape, size, and surface properties.

*L. aerocolonigenes* is a native producer of rebeccamycin, which is a natural product associated with not only antibiotics but also antitumoral properties. Nevertheless, high amounts of rebeccamycin are necessary for potential application in human treatments (Nettleton et al., 2019; Bush et al., 1987; Goel et al., 2003; Burstein et al., 2007; Schwandt et al., 2012; Pommerehne et al., 2019). As *L. aerocolonigenes* does not meet this requirement of high product titers naturally, a variation in morphology engineering techniques was applied to optimize rebeccamycin production in several recent studies. Supplementing glass macroparticles (100 g·L^−1^ glass beads with x_50_ = 969 µm) led to an increased rebeccamycin production of about an order of magnitude (Walisko et al., 2017; Schrader et al., 2019; Schrinner et al., 2020; Schrinner et al., 2021). Hereby, the application of glass beads induces defined mechanical stress to the culture broth and thus affects the cultivation in a profoundly different way than microparticles do by directly interacting with the hyphal network. Additionally, Schrinner et al. (2021) investigated the combined approach of macroparticle-enhanced cultivation (100 g·L^−1^ glass beads with x_50_ = 969 µm) of *L. aerocolonigenes* and the addition of the emulsifier soy lecithin (5 g·L^−1^). So far, this combined supplementation generated the highest rebeccamycin concentration of 388 mg·L^−1^ after 10 days of cultivation reported in the literature for the shake flask scale. In the present study, lecithin supplementation was revived and forwarded to an optimized lecithin setup (type and concentration) for shaking flask cultivations. The combination with MPEC was investigated for product formation and its effects on macromorphological aspects. Thereby, a divalent target was pursued to generally aim for easily achievable optimized product titers by combining beneficial morphology engineering techniques and to better understand them individually and collectively.

Furthermore, Walisko et al. (2017) used talc microparticles (*x* = 7 µm) in MPEC studies for *L. aerocolonigenes*, resulting in a three-fold increase in rebeccamycin concentration and altering the macromorphology compared to an unsupplemented control culture. Additionally, the application of surface-modified talc microparticles, which influenced the particles’ hydrophobicity and surface charge, revealed that surface properties play a crucial role in the interactions between particles and hyphae (Walisko et al., 2017). Similar investigations were earlier conducted by Etschmann et al. (2015) for further filamentous microorganisms, addressing a wide variance of microparticles and their properties being the reason for varying effects. Subsequently, it was proposed that the design of tailor-made particles with defined surface properties would help discriminate between potential causes and understanding the underlying MPEC effects (Etschmann et al., 2015). The exact particle properties necessary for enhanced product formation remain unclear and need to be better understood (Walisko et al., 2017). On this account, microparticles made of glass were chosen for MPEC in *L. aerocolonigenes* in this study as they were considered relatively stable, chemically and thermally, and also have the potential for surface modifications. Additionally, due to the ideal spherical shape of the synthesized glass beads, former variations of pellet shapes are reduced to a minimum only regarding variations in specific surface area due to particle size.

Furthermore, clarification is needed concerning the interrelationships between the inner pellet structure, oxygen concentration gradients in the pellet, and how microparticles affect those. Hille et al. (2005) introduced a technique to quantify fungal pellet structures. Confocal laser scanning microscopy (CLSM) combined with oxygen microelectrode measurements in the same samples have only been applied in pellets of *Aspergillus niger* so far. Hereby, the investigation of hyphal distributions within pellets helped describe the impact of the inner pellet structure on oxygen gradients in the mycelial structure. Those investigations also considered varying hydrodynamic conditions, as the measurements proceeded in a flow tube cell with adjustable hydrodynamic conditions. Furthermore, Gonciarz et al. (2016) applied oxygen microprofiling in pellets fixed in a Petri dish to investigate the impact of varying process conditions in bioreactor cultures of *Aspergillus terreus.* However, no profiling with pellets under microparticle supplementation was conducted, and the profiling within a Petri dish does not represent actual conditions during cultivation. Consequently, a further focus of this study was the transformation of the former described oxygen microprofiling technique for *A. niger* (Hille et al., 2005; Hille et al., 2009) to a system suitable for filamentous bacteria, such as *L. aerocolonigenes.* The upcoming challenges with prokaryotic systems are their more filigree hyphal structure and the overall reduced pellet size. Eventually, the edited technique was used to better understand the relationship between the inner pellet structure and oxygen supply within the pellets of the actinomycete *L. aerocolonigenes.*

## 2 Materials and methods

### 2.1 Strain and standard cultivation conditions

The filamentous bacterium *L. aerocolonigenes* DSM 44217 was purchased from the German Collection of Microorganisms and Cell Cultures (DSMZ, Braunschweig, Germany) and maintained as frozen biomass suspension at −80°C in glycerol (30% v/v).

Pre-cultures were inoculated with 1 mL of frozen stock solution and incubated for 2 days. The main cultures were inoculated by transferring 300 µL of pre-culture to another flask. Subsequently, the incubation was conducted for 10 days. Cultivation was performed in baffled (four) shake flasks (250 mL) on an orbital shaker (Certomat BS-1, Sartorius, Göttingen, Germany) in the dark (28°C, 50 mm amplitude, 120 min^−1^ shaking frequency) with 50 mL GYM (glucose–yeast–malt) medium (4 g·L^−1^ glucose, 4 g·L^−1^ yeast extract, and 10 g·L^−1^ malt extract, pH 7.2). Glucose and yeast–malt solutions were autoclaved separately at 121°C for 20 min and subsequently mixed. All experiments were conducted in triplicate if not stated otherwise. Shaking flasks [four baffles, maximum inner diameter (d_sf_) of 81.3 mm] were of the same design for all experiments to minimize influencing the hydrodynamic conditions by varying flask geometry (Schrader et al., 2019; Schrinner et al., 2021).

MPEC experiments were performed by the addition of talc microparticles (talc powder, 10 μm, Sigma-Aldrich GmbH, Darmstadt, Germany) or soda-lime glass microparticles (SOLID Micro Glass Beads, type S, x = 0–20 and 0–50 µm) purchased from Sigmund Lindner GmbH (Warmensteinach, Germany). Particle size analysis was performed experimentally by laser diffraction (Mast and Stegmann 2019, Malvern Panalytical GmbH, Kassel, Germany). Particle sizes were estimated to be x_50_ = 7.9 µm (x_10_ = 0.9 µm, x_90_ = 16.3 µm) and x_50_ = 30.5 µm (x_10_ = 15.0 µm, x_90_ = 50.5 µm), respectively. In the following the microparticles will be denoted as their determined median particle size. For cultivation with microparticle supplementation, varying amounts were added to shaking flasks at certain weight concentrations and suspended in 10 mL MilliQ^®^ water before autoclaving. After sterilization, 40 mL of accordingly concentrated, sterile GYM medium was added to the shaking flasks. At the same time, the subsequent inoculation with pre-culture and incubation for 10 days were performed similarly to unsupplemented standard cultivation.

In one experiment, soy lecithin (EMULPUR™ IP, Cargill Texturizing Solutions Deutschland GmbH and Co., KG, Hamburg, Germany) was investigated as a further supplement in *L. aerocolonigenes* cultivations. A stock solution of 75 g·L^−1^ lecithin (separately sterilized) was prepared and further diluted with MilliQ^®^ water. A final concentration of 7.5 g·L^−1^ in the shake flask was set by adding 40 mL of accordingly concentrated GYM medium to obtain a final volume of 50 mL. MPEC, in combination with lecithin, was prepared by adding the microparticles, the lecithin stock solution, and MilliQ^®^ water into the shake flask before autoclaving.

### 2.2 Rebeccamycin and cell dry weight quantification

Either 10 or 20 mL of cultivation broth was transferred to 50-mL experimental tubes and used for rebeccamycin extraction by adding 5 mL ethyl acetate. Afterward, incubation in an overhead shaker (Intelli-Mixer RM-2 M, LTF Labortechnik, Wasserburg, Germany) for 60 min at room temperature (RT) was initiated. Subsequently, the samples were centrifuged (RT, 4,000 min^−1^, 10 min; Heraeus Varifuge 3.0R, Thermo Fisher Scientific, Waltham, United States), and the ethyl acetate phase was removed for analysis *via* HPLC as described previously by Schrinner et al. (2020).

Cell dry weight concentration (CDW) was determined gravimetrically by vacuum filtration with filter papers (Quantitative Papers/Grade 369, Sartorius, Göttingen, Germany) in duplicate. As the supplemented microparticles were also detained by filtration, the estimated CDW would be enhanced significantly. Thus, an alternative method was developed during experiments and applied to eliminate the particle influence when stated. After vacuum filtration with pre-weighted filters, the filter cake was carefully washed with MilliQ^®^ water in a Petri dish to remove particles, which are loosely attached to the biomass. The supernatant of the washing procedure and the filters with remaining biomass were collected separately in pre-weighted ceramic crucibles, dried (105°C, 48 h), and cooled to RT in a desiccator (30 min), and the crucible weights were determined. The crucibles were transferred to a pre-tempered muffle furnace (Heraeus M110, Fisher Scientific GmbH, Schwerte, Germany) and incubated for 130 min at 550°C to incinerate the biomass and filter. Again, the weights of the crucibles were gravimetrically determined after cooling to RT in the desiccator. By considering the weight of the ashes negligible, the weight differences after every step of the procedure break down the isolated weight of biomass and microparticles. Thus, the concentration of microparticles embedded in the biomass can also be derived indirectly (the negligible weight of the ashes is assigned to the microparticle’s weight).

### 2.3 Investigation of pellet characteristics

Pellet size distributions were measured by laser diffraction (Mastersizer 2000; Malvern Panalytical GmbH, Kassel, Germany). For pellet imaging, a digital inverse microscope (EVOS XL, AMG, Bothell, WA, United States) was used to take at least 20 images to ensure statistical reliability. Morphology analysis was conducted using the software Fiji/ImageJ (National Institute of Health, Bethesda, United States), whereby the area-equivalent spherical diameter (AESD) (Eq. 1) was the morphological parameter of interest and is defined as follows:

$$AESD=\sqrt{\frac{4∙Area}{\pi }}.$$ (1)

### 2.4 Pellet slicing, viability staining, and CLSM

For an investigation of the inner pellet structure, a pellet slicing technique (Lin et al., 2010; Krull et al., 2013; Dinius et al., 2023) was applied to obtain pellet cross-sections. Initially, single pellets were isolated and located in the frozen section medium (Richard-Allan Scientific Neg-50 Frozen Section Medium, Thermo Fisher Scientific, Waltham, United States). On a sample holder, freezing time was shortened using a rapid freezing station in a cryostat microtome (HM 550, Microm, Neuss, Germany). Defined slices of 100 µm were cut with a fixed blade inside the cryochamber. Pellet slices were transferred to microscope slides, and images of inner cross sections were captured using a microscope (Evos XL, AMG, Bothell, WA, United States).

To monitor the pellet viability during cultivation, viability staining was applied as previously described in detail (Schrinner et al., 2020). Shortly, staining proceeded with 1.5 µL SYTO9 (3.34 mM) and 1.5 µL propidium iodide (PI, 20 mM) (Molecular Probes, Eugene, United States) in 1 mL of 1x salt solution (0.31 g·L^−1^ ammonium chloride, 4.33 g·L^−1^ disodium phosphate, 0.13 g·L^−1^ potassium chloride, and 3.04 g·L^−1^ sodium dihydrogen phosphate dihydrate). Single pellets were transferred to the staining solution and incubated for 15 min in darkness. Cross-sections of the pellets were sliced and investigated using a confocal laser scanning microscope (CLSM, C2si, Nikon Instruments, Amsterdam, Netherlands). Living parts of the pellets were detected with a 510- to 540-nm laser showing green fluorescence due to staining with SYTO9. Dead parts were detected with a 620- to 650-nm laser as a red fluorescence signal due to PI staining. Both stains are nucleic acid intercalating agents. SYTO9 permeates cell membranes, staining all cells (live and dead). PI, which is impermeable for cell membranes, only stains cells with non-intact membranes. Due to a higher affinity toward nucleic acids, PI replaces SYTO9 when both stains are present (Stocks, 2004). The software Fiji/ImageJ 1.52 h (National Institute of Health, Bethesda, United States) was used for image analysis. A plugin voxel counter was applied to determine the number of living (green) and dead (red) parts of the pellets. The live ratio of pellets was calculated as follows:

$$Live\;ratio=\frac{Green\;pixels}{Green\;pixels+Red\;pixels}.$$ (2)

### 2.5 Elucidation of pellet densities

A gravimetric approach, previously described by Hille et al. (2009), was applied to estimate pellet densities. For each MPEC approach (0, 2.5, 5, 7.5, and 10 g·L^−1^ glass microparticles), 80–200 pellets of similar size were selected (x_Pellet_ ± 200 μm, minimum 500 µm) and subsequently microscopically investigated (see Section 2.3) to calculate their wet volume. Pellets were washed three times with 2 mL deionized water in a sampling tube, and CDW was determined (see Section 2.2). The specific pellet density ρ is defined as dry mass (m_d_) per wet volume (V_p_):

$$\rho =\frac{m_{d}}{V_{p}}.$$ (3)

Second, a negative staining CLSM method described by Hille et al. (2005) was modified and applied to investigate hyphal density, in which the intercellular area between the hyphae is stained instead of the hyphae themselves. After oxygen microprofiling (see Section 2.6), the pellets were transferred to a frozen section medium, and slices of 100-µm thickness were cut (see Section 2.4). Slices were incubated with 3 µL fluorescence-labeled dextran (1 mg·L^−1^, Dextran, Fluorescein, 10,000 MW, Anionic, Life Technologies GmbH, Darmstadt, Germany) for 15 min (RT, dark) before CLSM (C2si, Nikon Instruments, Amsterdam, Netherlands) measurements were conducted. For fluorescence detection, laser extinction was set to 488 nm, and the emission range was set to 500–550 nm. To obtain better comparability among single pellets, a threshold was defined for the background signal and multi-layered imaging to a depth of 50 µm was conducted.

### 2.6 Oxygen microprofiling

**FIGURE 1:**
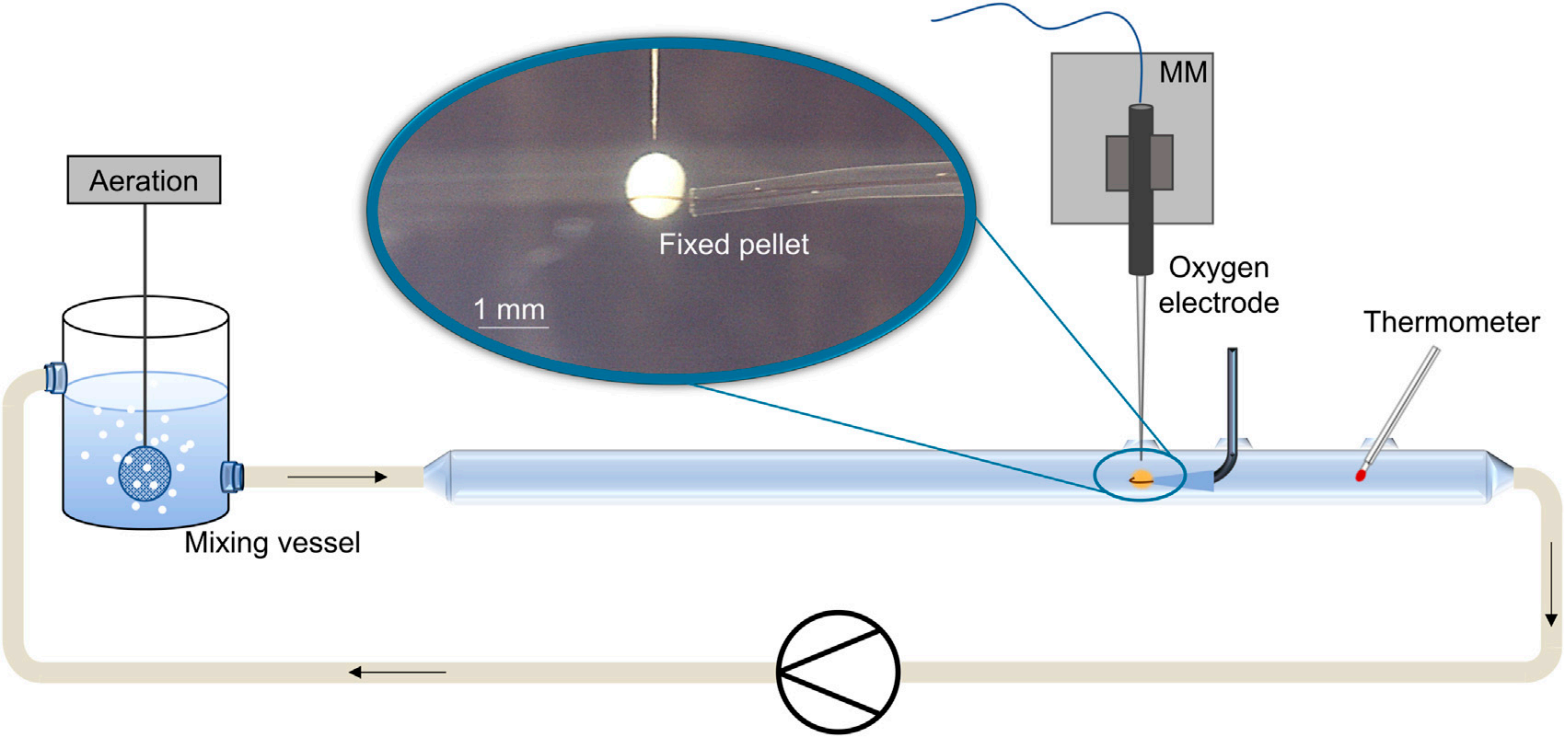
Scheme of the experimental setup for oxygen microprofiling in biopellets (blue encircled close-up) of filamentous microorganisms. Pellet analysis occurs in a glass tube (laminar flow conditions) with the help of oxygen micro-sensors fixed on a software-controlled micromanipulator (MM). Oxygen concentration is measured in 10 µm step sizes inside the pellet.

For oxygen microprofiling, the setup described by Hille et al. (2005) was updated (Figure 1). Devices and accessories were purchased from Unisense A/S (Aarhus, Denmark) and included a Clark-type oxygen microsensor (Oxygen Microsensor 10 μm, Ox-10) with a maximum tip diameter of 10 µm for minimal invasive pellet penetration, which is fixed on a micromanipulator (MM) (MM33-M) on top of a motorized micromanipulator stage (MMS) with adapter set (MMS-A) and is controlled by a motor controller (MOTCON). Furthermore, a picoammeter (PA 2000), a two-channel A/D converter (ADC 216), and the control software (SensorTrace Suite v3.3.175) were used for data acquisition. A 1.7-m-long glass pipe (inner diameter 24 mm) was used as a flow cell and was placed horizontally on a stable mounting. For temperature monitoring, a thermometer was installed. Silicon tubes connected the pipe with a magnetic gear pump for well-defined laminar flow conditions and with an aerated (compressed air) or non-aerated (nitrogen) preheated mixing vessel. Pellets were fixed in a loop of a single human hair at the tip of a gel-loading tip fixed on a mounting in the center of the flow cell. Subsequently, the microsensor was placed directly above the pellet, and the pellet surface was determined by camera (board-level camera) visualization and detected an abrupt oxygen decrease. Controlled by the software, the sensor was driven stepwise (10 µm each step) into the pellet at the equator area of the pellet, while the local oxygen concentration was measured with time and spatial resolution perpendicular to the flow direction in the tube. A 20% dilution of a minimal alternative medium (50 g·L^−1^ maltose, 21 g·L^−1^ MOPS, 8.8 g·L^−1^ glutamate, 0.2 g·L^−1^ MgSO_4_∙7H_2_O, 9 mg·L^−1^ FeSO_4_∙7H_2_O, 1 mg·L^−1^ CaCl_2_, 1 mg·L^−1^ NaCl, 15 mL K_3_PO_4_ solution, and trace elements, pH 7.2) for *L. aerocolonigenes* was used, as the complex GYM medium leads to the fast growth of unwanted microorganisms within the non-sterile flow system, which influences the oxygen supply. The temperature of the fluid was set to a cultivation temperature of 28°C. Before measurements, the pellet rested for 30 min in the flow cell to adjust to the ambient pseudo-steady-state conditions. A minimum of three pellets was investigated per microparticle concentration, and at least three oxygen profiles were recorded for each pellet.

### 2.7 Statistical analysis

Single-factor analysis of variance (ANOVA) was conducted to clarify if glass microparticles significantly enhance rebeccamycin concentration compared to control cultivation and talc-supplemented approach. The statistical test (*α* = 0.05) was performed in Microsoft Excel. A *p*-value lower than 0.05 (an F-value higher than the critical F-value, respectively) represents a statistically significant difference between the two means. Due to the generally small sample number (duplicates or triplicates), no further data were analyzed (possible type II errors).

## 3 Results and discussion

In this study, several aspects regarding the supplementation of microparticles and the addition of the emulsifier soy lecithin in the course of MPEC for the filamentous bacterium *L. aerocolonigenes* were systematically investigated. The attention was especially drawn to the aspects of altering productivity going hand in hand with changes in cellular morphology.

### 3.1 Influence of varying microparticle materials on product formation

**FIGURE 2:**
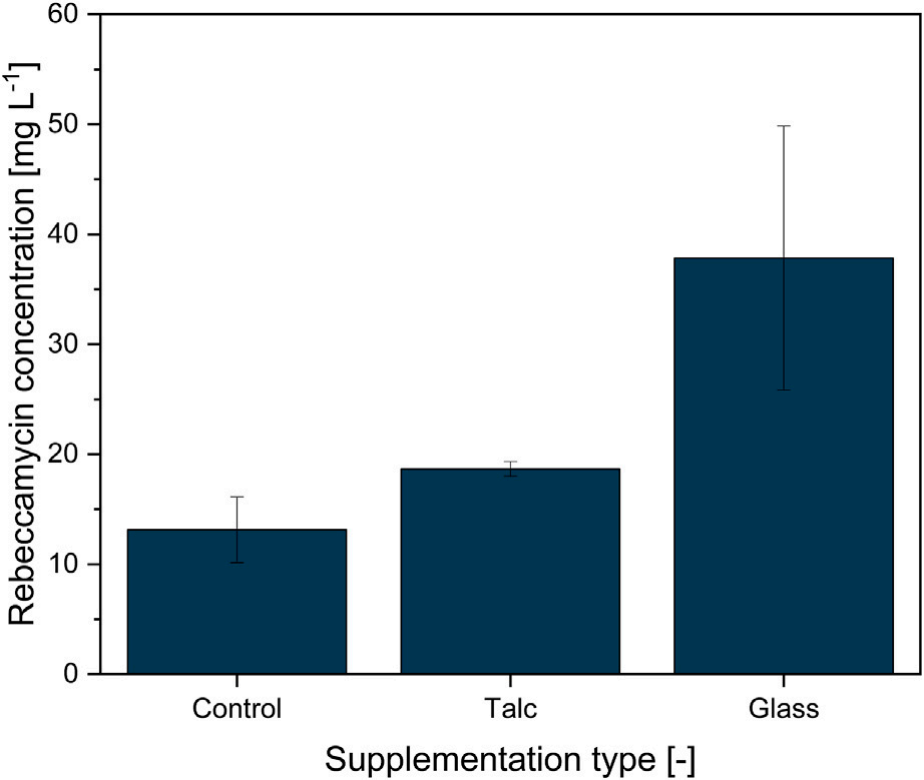
Rebeccamycin concentration of 10-day *L. aerocolonigenes* cultivation without microparticle addition (control) compared to cultivation with talc microparticle addition (10 g·L^−1^, 10 µm) and glass microparticle addition (10 g·L^−1^, x_50_ = 7.9 µm).

An earlier study by Walisko et al. (2017) showed that MPEC generally impacted the product formation of the filamentous bacterium *L. aerocolonigenes* in shake flask cultures. Based on these results, an initial study was conducted to determine the suitability of glass particles with defined properties for MPEC of *L. aerocolonigenes* and how they perform compared to conventionally used talc microparticles*.* For this study, a concentration of 10 g·L^−1^ for both microparticle types, talc (x = 10 µm) and soda-lime glass (x_50_ = 7.9 µm), was chosen, as this is a common choice in the medium concentration range for various microparticle types and organisms discussed throughout the literature (Driouch et al., 2010b; Walisko et al., 2017). The resulting rebeccamycin concentration of 10-day *L. aerocolonigenes* cultivation of an unsupplemented control culture compared to MPEC batches with talc and glass microparticles is depicted in Figure 2.

Without microparticle addition to the culture, the rebeccamycin titer had a value of only 13.1 mg·L^−1^, whereas the titer increased to 18.7 mg·L^−1^ in the presence of talc (+43%). Considering the results of similar cultivation by Walsiko et al. (2017), the titer of rebeccamycin lies in the range of control cultures, whereas supplementation with talc microparticles leads to clearly lower product titer. A direct comparison of product titers is not necessarily reasonable as cultures vary significantly between isolated biological approaches. In defiance of a relatively high standard deviation for the glass microparticle batch, a maximum titer of 37.8 mg·L^−1^ (+286%) was reached. The statistical analysis confirmed that the differences between the cultivation were statistically significant (*p* = 0.0131). Thus, glass microparticles showed a considerable advantage over talc microparticles and were used for further investigations.

### 3.2 Microparticle concentration and size

**FIGURE 3:**
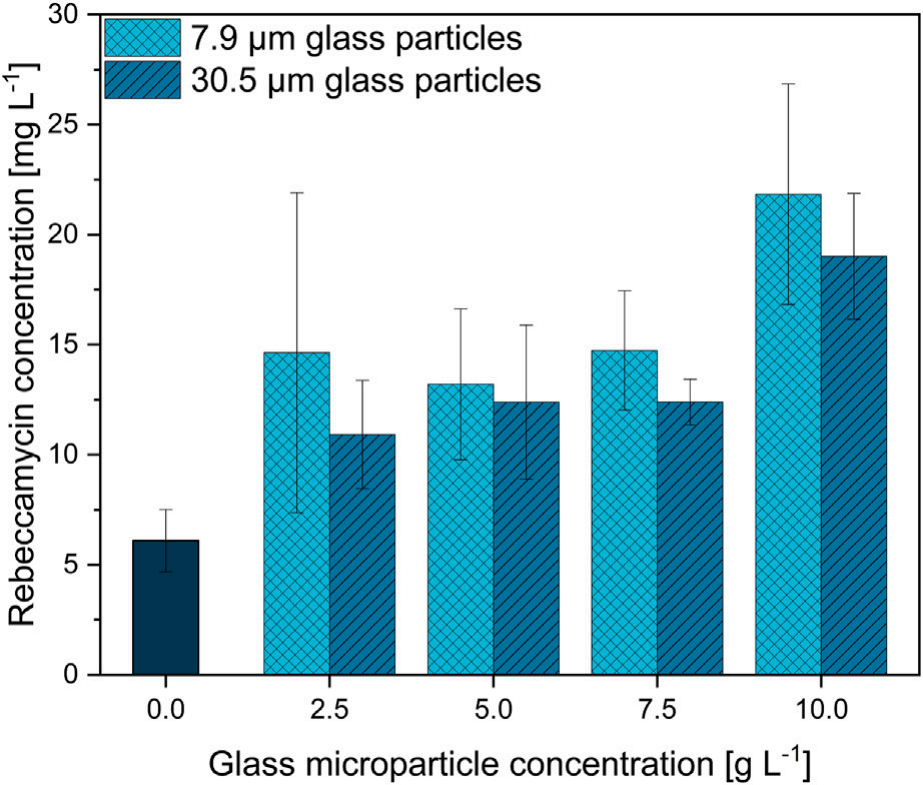
Rebeccamycin concentration of 10-day *L. aerocolonigenes* cultivation without microparticle addition (control) compared to MPEC with glass microparticle addition (x_50_ = 7.9 or 30.5 µm) in varying concentrations in the range of 2.5–10 g·L^−1^.

The previous section indicated 10 g·L^−1^ to be a reasonable concentration for glass microparticle supplementation in *L. aerocolonigenes*. Nevertheless, the adequate concentration and microparticle setup broadly vary throughout the literature as it is highly specific for each organism and each microparticle type. Therefore, in order to select the best microparticle setup and effects on rebeccamycin production, glass microparticles were screened in the concentration range of up to 10 g·L^−1^ and in two different particle sizes (x_50_ = 7.9 and x_50_ = 30.5 µm) during *L. aerocolonigenes* cultivations. The resulting product titers of 10-day cultivation for the control culture (no microparticle addition) and for the glass microparticle concentrations of 2.5, 5.0, 7.5, and 10 g·L^−1^ are presented in Figure 3.

In comparison to the unsupplemented control culture resulting in a rebeccamycin titer of 6.1 mg·L^−1^, two clear trends can be observed regarding rebeccamycin concentration as a function of glass microparticle addition. An increasing glass microparticle concentration tends to enable the production of enhanced rebeccamycin titer. As the addition of 2.5, 5.0, and 7.5 g·L^−1^ microparticles elevates the product titer to a more or less stable concentration plateau, a further increase in microparticle concentration increases the product concentration to a maximum value of 21.8 mg·L^−1^ for 7.9-µm glass microparticles and 19 mg·L^−1^ for 30.5-µm glass microparticles. The resulting increases in product concentration are 357% and 311%, respectively. Although the overall difference is small, the product titers are always slightly higher for the cultivation with a smaller 7.9-µm microparticle fraction.

**FIGURE 4:**
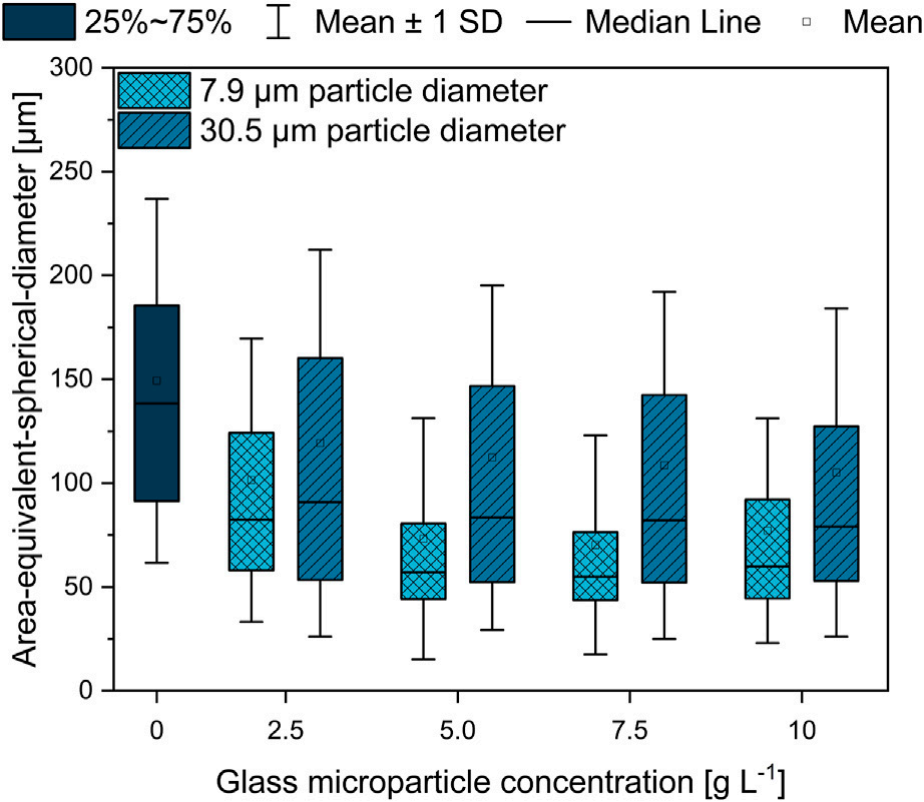
Area-equivalent spherical diameter (AESD) of *L. aerocolonigenes* pellets from 10-day cultivation without glass microparticle addition (control) compared to MPEC with glass microparticle sizes of x_50_ = 7.9 or 30.5 µm in varying concentrations in the range of 2.5–10 g·L^−1^.

On the basis of the broadly described effect of MPEC on the organism’s macromorphology in the literature (Kaup et al., 2008; Driouch et al., 2010b), the focus here was set on the pellet morphology changes induced by glass microparticles on *L. aerocolonigenes*, which is illustrated in the form of the AESD of pellets for each discussed glass microparticle concentration of both sizes (Figure 4).

Two conclusions can be drawn. In comparison to the unsupplemented control culture, both MPEC approaches lead to lower AESD, whereas for all glass microparticle concentrations, the difference is more distinct in the case of smaller glass microparticles. Furthermore, with increasing microparticle concentration, a tendency of decreasing pellet sizes can be observed, whereby mean pellet sizes of about 110 µm (in the presence of 30.9 µm glass microparticles) and 75 µm (7.9 µm glass microparticles) are approximated by microparticle addition.

**FIGURE 5:**
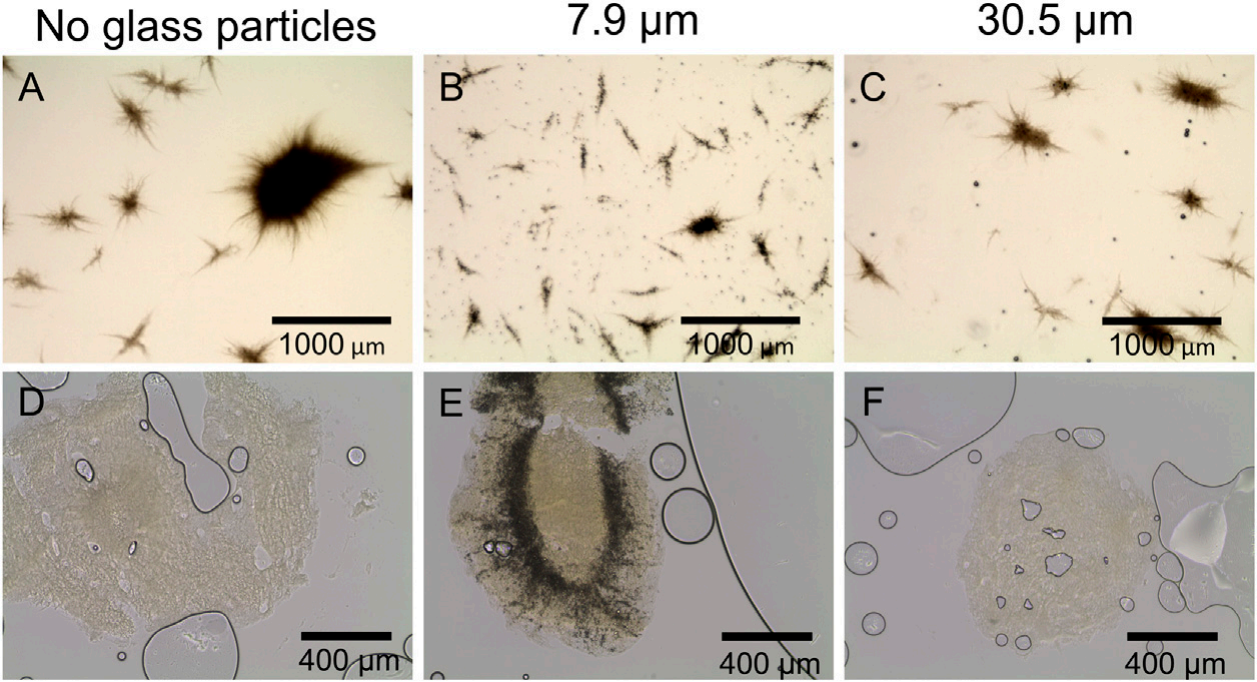
Microscopic images of *L. aerocolonigenes* **(A)** without and **(B, C)** with glass microparticle addition (10 g L^-1^, x_50_ = 7.9 and 30.5 µm) and **(D–F)** of pellet slices of the same microparticle setup. Here, black lines and circles are air bubbles enclosed in the sectioning medium. Dark areas in the pellets are incorporated glass microparticles.

Accordingly, in Figure 5, microscopic images of the corresponding control cultures (Figure 5A, no glass microparticles) and of maximum particle concentration of 10 g·L^−1^ (Figure 5B, x_50_ = 7.9; Figure 5C, x_50_ = 30.5 µm) show distinct pellets to still be the majority among culture broth apart from few clumps or mycelial structures. Such observations match the findings in earlier studies. For instance, Kuhl et al. (2020) observed a pellet size decrease in a similar range when cultivating the filamentous bacterium *Streptomyces albus* with similar talc microparticle (10 µm) concentrations. Moreover, several investigations led to fungal pellets decreasing in size due to MPEC (Driouch et al., 2010b; Gonciarz et al., 2016; Yatmaz et al., 2020). By reconsidering the corresponding rebeccamycin concentration, the results are in good accordance, as smaller pellets are associated with enhanced product formation due to better oxygen transfer into the pellets and fewer areas of oxygen limitations, as previously discussed. Consequentially, although the decrease in the pellet size is an important key for enhanced rebeccamycin formation, further effects caused by microparticles should be considered, as further described in this study.

Generally, MPEC is associated with the entanglement or incorporation of microparticles into the micromorphological pellet structure (Driouch et al., 2012; Walisko et al., 2015), which was the main subject of the subsequent investigations of pellet cross sections with a slide thickness of 100 µm (Figure 5D–F). In Figure 5D or Figure 5F, the pellet slices only consist of biomass, which is the light, slightly structured area with few scratches and gaps originating from the slicing process. Larger black circles are air bubbles enclosed in the sectioning medium. However, Figure 5E shows a broad incorporation of glass microparticles into the pellets, indicated by the dark area encircling the pellet center and proceeding radially to the surface. Similar incorporation behavior was observed for pellets of the remaining microparticle concentrations (data not shown). It can be concluded that microparticle incorporation only occurs to a significant extent when the smaller microparticles (x_50_ = 7.9 µm) are present in the culture. Furthermore, the cross-sections indicate the amount of incorporated microparticles to be increased with increasing particle concentrations. These results assume particle incorporation only occurs when a certain microparticle size is not exceeded. As mentioned, microparticle incorporation is a phenomenon described by several authors, mostly observed by conventional microscopy. Within the presented innovative cross-sectional images of filamentous bacteria originating from MPEC, it can be assumed that incorporation does not randomly occur within the whole biomass pellet but according to certain patterns, e.g., a ring encircling the pellet core. Similar results were obtained by Driouch et al. (2012), showing the incorporation of varying microparticles into the pellet centers of fungal *A. nige*r pellets (core-shell pattern). Consequentially, the same mechanism is assumed to lead to an increase in product formation with increasing microparticle concentration—incorporated microparticles may be a factor for a reduced pellet density, resulting in an enhanced supply of nutrients (in most cases, oxygen) and the generation of more active biomass. However, in the case of the cultivation approaches with bigger glass microparticles (x_50_ = 30.5 µm) being supplemented to the culture, this hypothesis is questionable. As the supplementation nevertheless leads to enhanced rebeccamycin titers, although no particles were incorporated into the biomass, it can be theorized that other mechanisms may take hold. A variety of possible surface-chemistry effects (e.g., ion leaching or adsorption effects) are described in Laible et al. (2021) and will be focused on in future studies.

### 3.3 Growth kinetics and cell viability

**FIGURE 6:**
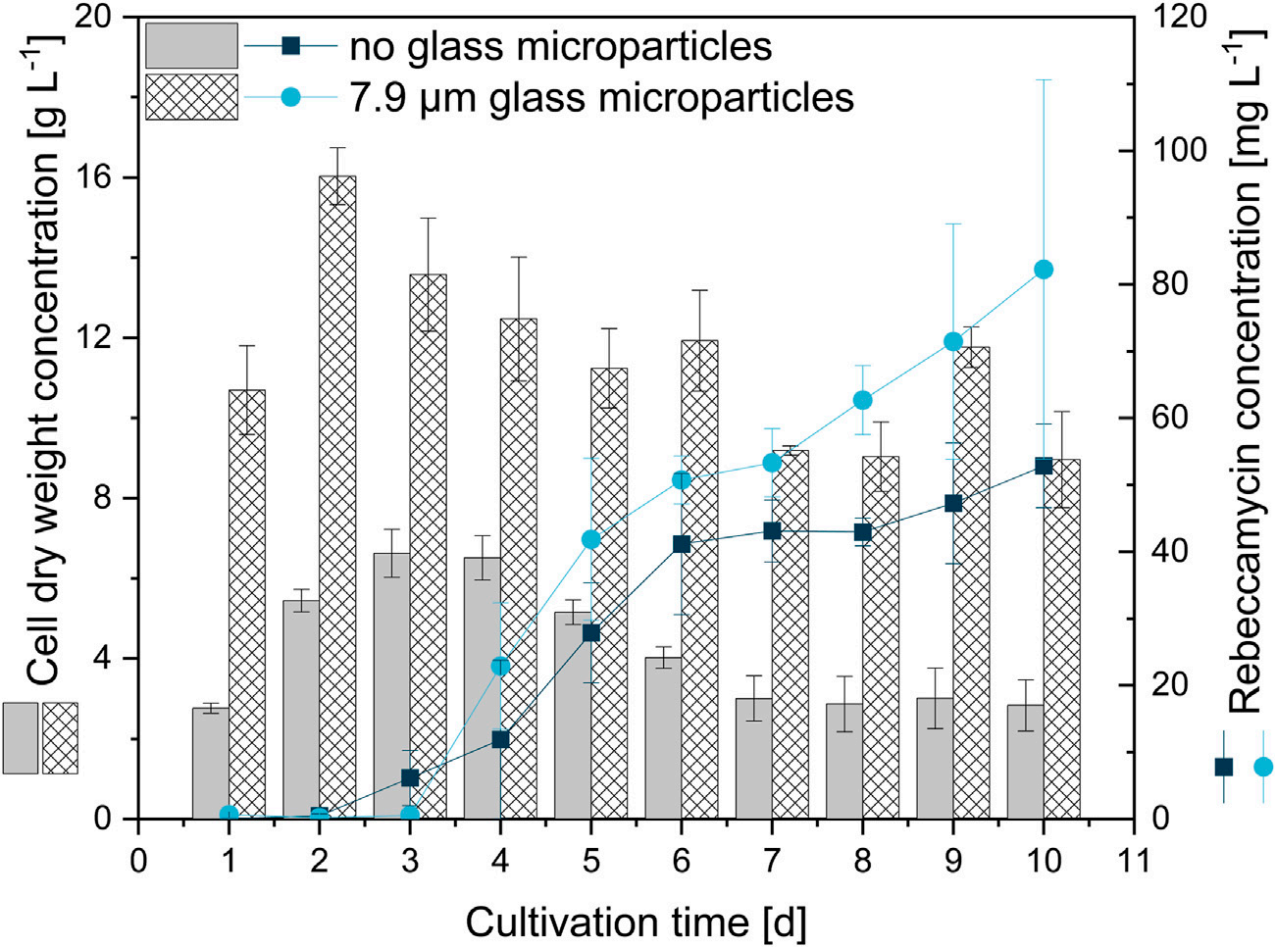
Cell dry weight concentration and rebeccamycin concentration for 10-day *L. aerocolonigenes* cultivation without glass microparticle addition (control, grey, and dark blue circles) compared to MPEC with glass microparticles (10 g·L^−1^, x_50_ = 7.9 µm white patterned, light blue squares).

For a better understanding of the underlying effects leading to enhanced product formation, further studies were performed to obtain a time resolution of the occurring differences between control and microparticle-supplemented culture (x_50_ = 7.9 µm) during 10-day cultivation (Figure 6). Cell dry weight concentrations of the microparticle-supplemented culture appear to be significantly higher than the CDW of the control culture. The values represent the CDW and weight of microparticles incorporated into the biomass and/or remaining in the supernatant, as both biomass and microparticles were retained by filters. Thus, the general trend should draw more focus than the absolute values, in which both approaches show similar CDW trends. While the CDW of the glass microparticle-supplemented approach peaks after 2 days of cultivation, a delayed maximum CDW is reached after 3 days of cultivation for the unsupplemented control. Both CDWs decrease thereafter until reaching a threshold. The CDW trend also correlates with the glucose concentrations (data not shown). While glucose is completely depleted after 2 days in MPEC, this is the case after 3 days for the control culture. As rebeccamycin is a secondary metabolite, its non-growth-associated product formation is detected after glucose depletion. This is the case between days two and three for the unsupplemented control and slightly delayed on day three for the microparticle-supplemented culture, although the microparticle-supplemented culture reached the maximum CDW earlier. However, the final rebeccamycin concentration after 10 days for the MPEC approach is significantly higher and reaches 82 mg·L^−1^ in contrast to the 53 mg·L^−1^ for the unsupplemented control. Additionally, AESD was analyzed daily for both cultures (data not shown). Again, a significant reduction in pellet size was obtained, especially for the first 5 days of cultivation, accompanied by a rather narrow pellet size distribution. The assumption can be made that the reduction in pellet size over the whole cultivation time and, thus, a better oxygen supply could be a factor for overall increased product synthesis.

**FIGURE 7:**
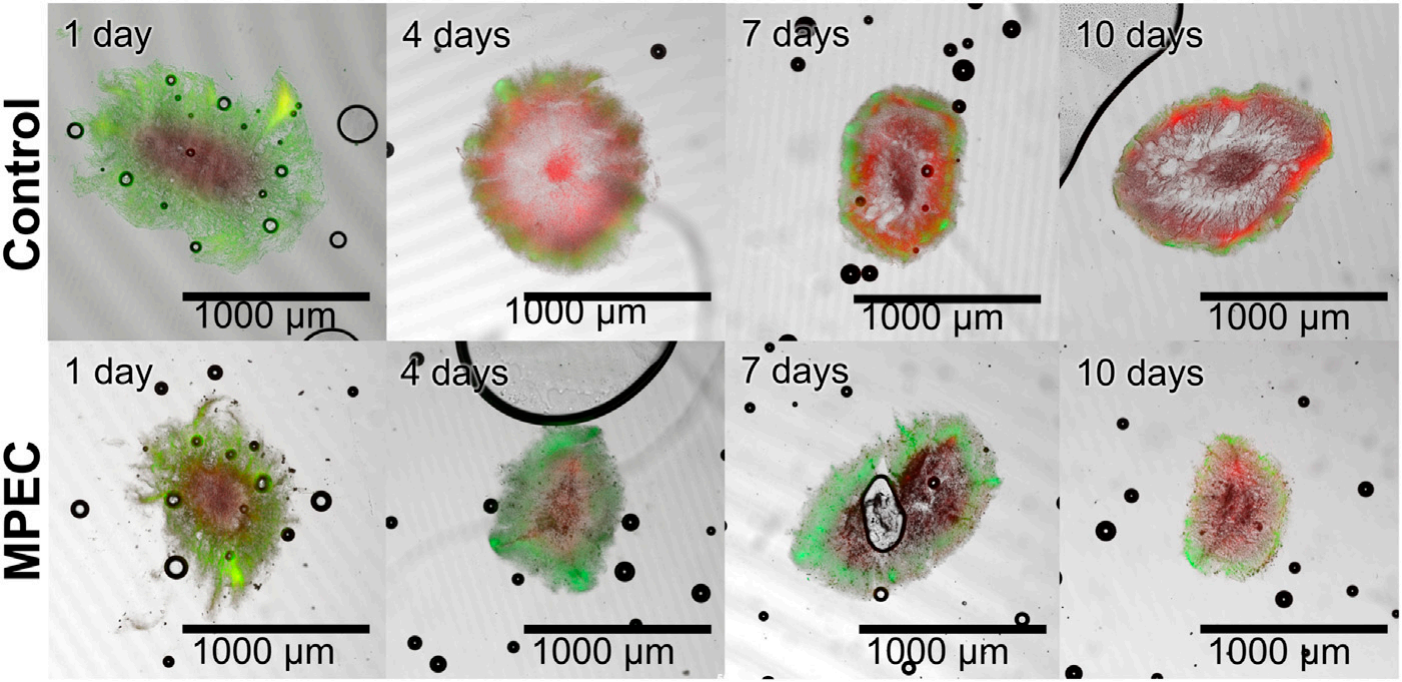
Exemplary microscopic images of sliced pellets from unsupplemented cultivation (control) (upper row) and a glass microparticle-supplemented cultivation (10 g·L^−1^, x_50_ = 7.9 µm) (lower row) 10-day *L. aerocolonigenes* cultivation obtained with CLSM. Staining was performed with SYTO9 (green fluorescence, living cells) and PI (red fluorescence, dead cells).

For a further evaluation of the effects of glass microparticles on the pellets, this study also focused on the metabolic activity of cells within the pellets in the form of their viability. Therefore, viability staining, subsequent pellet slicing, and CLSM measurements were conducted for pellets selected on a daily sampling basis during cultivation. A selection of the obtained CLSM images is presented in Figure 7. After 1 day of cultivation, the images show similar staining of the pellets, as the control and the MPEC pellet both show extended green (viable, oxygen-supplied) areas. In the course of the unsupplemented control cultivation, the red (dead) parts in the pellet predominate quickly and leave only minor parts of viable cells already on day four. The share of dead parts is generally increasing with increasing cultivation time. However, the cultivation with glass microparticles shows a more stable course in regard to viable cells over the whole cultivation time. It can be clearly visually seen that overall, the extent of dead areas is delayed significantly.

**FIGURE 8:**
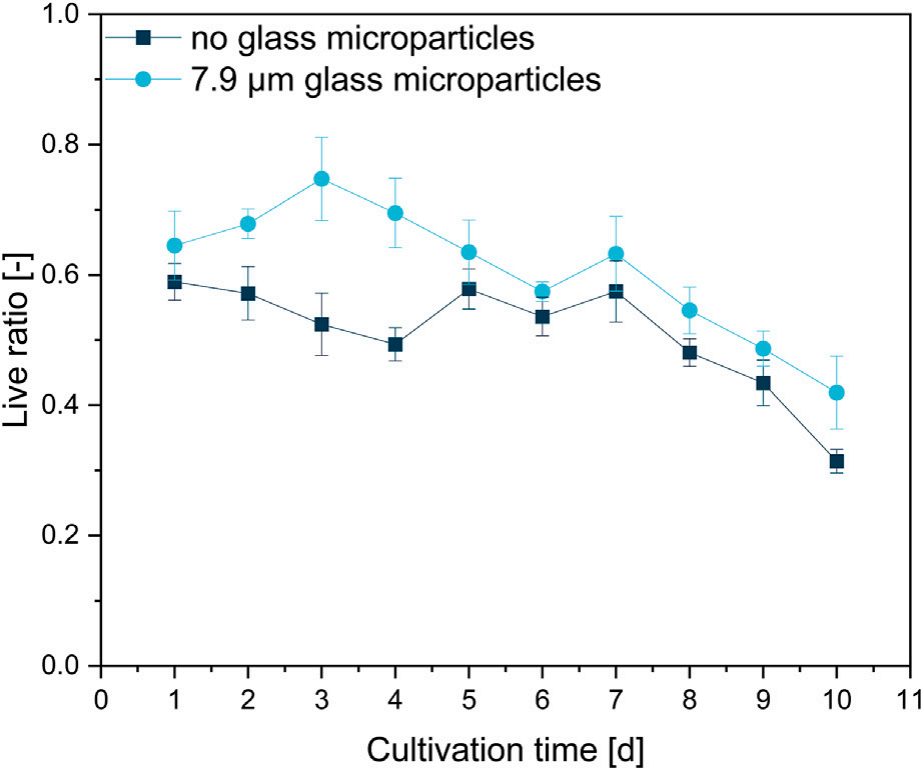
Live ratio calculated with Eq. 2 of fluorescently stained pellet slices originating from unsupplemented control cultivation and a glass microparticle supplemented MPEC (10 g·L^−1^, x_50_ = 7.9 µm) cultivation of *L. aerocolonigenes*.

This rather qualitative evaluation can also be confirmed by the course of the viability obtained by image analysis (Figure 8). The live ratio of the MPEC approach is overall increased compared to the control culture; significant differences are visible, especially between days one and four. The live ratio of the supplemented culture reaches a maximum (0.75) on day three, followed by a steady decrease and a minimum live ratio of 0.42 after 10 days of cultivation. For the control culture, the live ratio decreases slightly until day four (exponential growth and initial stationary phase). Subsequently, after a steady-state phase, a significant decrease results in a final live ratio of 0.31. Such a decrease is connected to the biomass death phase, which leads to cell lysis and can also partly be identified in the form of the patchy biomass distribution in several cross-sections in Figure 7.

Pellet slicing and subsequent CLSM are time-consuming, extensive manual procedures, and far from statistically high-throughput reliability. The presented results, for instance, are based on six pellet slices per day for each approach. However, the presented results describe a trend of the effects on viability by glass microparticle addition. A similar effect was observed by Driouch et al. (2010a), in which a GFP-producing *A. niger* strain was cultivated without and with 5 g·L^−1^ talc microparticles, which led to growth in the form of pellets and mycelium, respectively. Although the pellet only showed a thin outer layer of GFP production, the mycelium resulting from particle supplementation showed extensive GFP production over the entire mycelium. Moreover, a recent study by Schrinner et al. (2020) showed glass bead macroparticle addition (100 g·L^−1^, x_50_ = 969 μm) to be beneficial for the higher viability of cells over time in comparison to an unsupplemented control culture. Moreover, this parallel is further enhanced by the observation that these pellets of higher viability also showed enhanced rebeccamycin production. As already concluded by Schrinner et al. (2020), it is evident that a larger proportion of living cells leads to higher product synthesis. The supplementation with microparticles leads to decreased pellet sizes and, thus, a better supply of nutrients and oxygen into the pellets (Driouch et al., 2010b; Gonciarz and Bizukojc, 2014; Walisko et al., 2017). However, Figure 7 clearly indicates that the pellet size (as the investigated pellets are in the same size range) is not the only factor. The incorporation of the microparticles into the *L. aerocolonigenes* pellets suggests that an additional loosening of the pellet structure by microparticles entangled between single hyphae occurs. Such a decrease in overall pellet density, again, contributes to a better supply of nutrients and oxygen into deeper pellet layers, which is investigated in Section 3.5 and Section 3.6.

### 3.4 MPEC combined with soy lecithin supplementation

Schrinner et al. (2021) investigated the effect of soy lecithin supplementation on the cultivation of *L. aerocolonigenes*, also in combination with glass bead macroparticle addition (100 g·L^−1^, x_50_ = 969 μm). The presented results showed that lecithin is highly beneficial for rebeccamycin production in both cases, as a single supplement and in combination with macro-sized glass beads. Following that, investigations were conducted to investigate the combination of lecithin and microparticles and how biomass is affected in this context.

**FIGURE 9:**
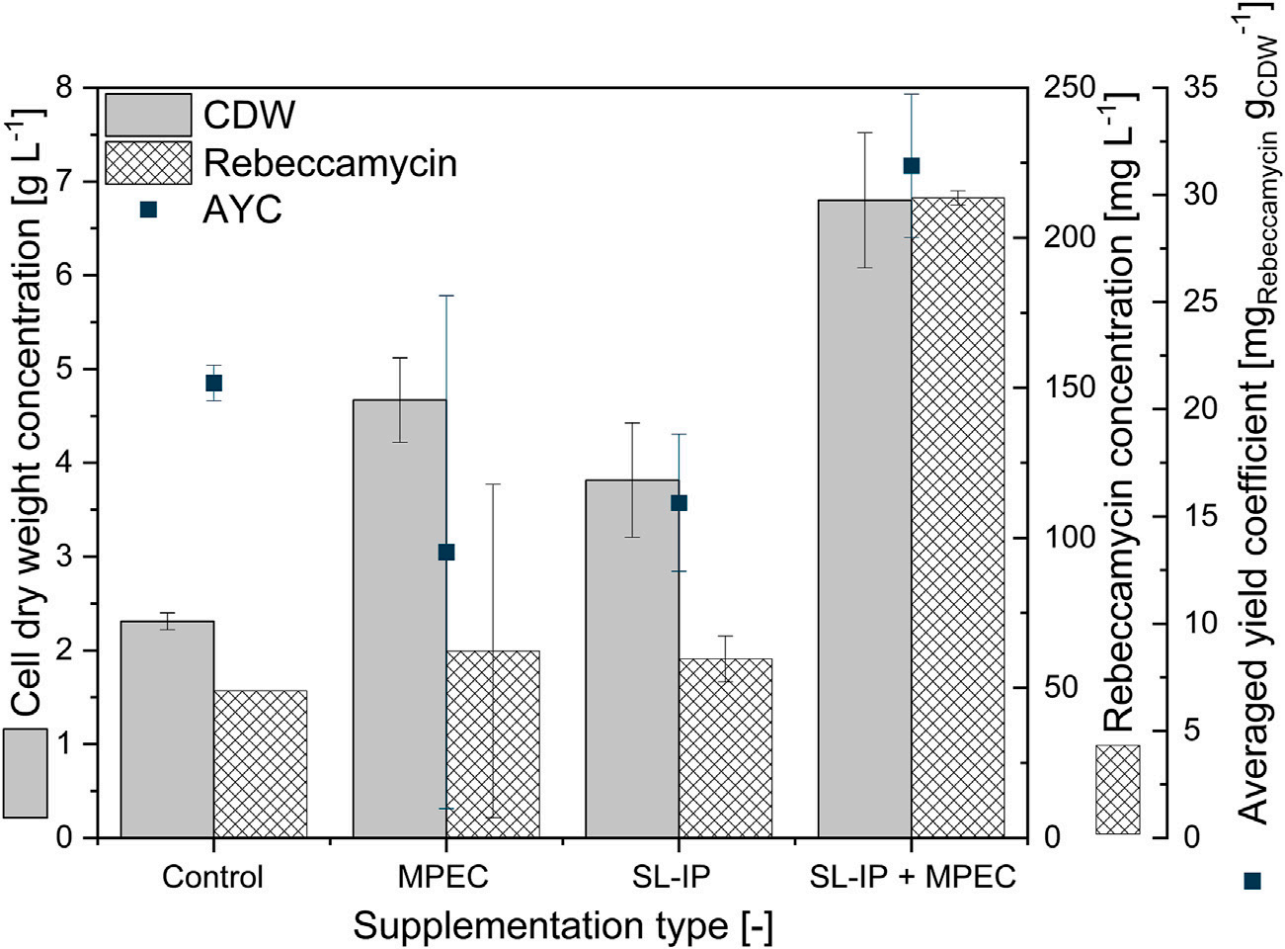
Cell dry weight concentration, rebeccamycin concentration, and averaged biomass-related product yield coefficient (AYC) of 10-day *L. aerocolonigenes* cultivation of an unsupplemented control culture, MPEC supplemented with glass microparticles (10 g·L^−1^, x_50_ = 7.9 µm), soy lecithin-supplemented culture (SL-IP, 7.5 g·L^−1^) and MPEC supplemented with glass microparticles (10 g·L^−1^, x_50_ = 7.9 µm) combined with soy lecithin addition (SL-IP, 7.5 g·L^−1^) (SL-IP + MPEC).

**FIGURE 10:**
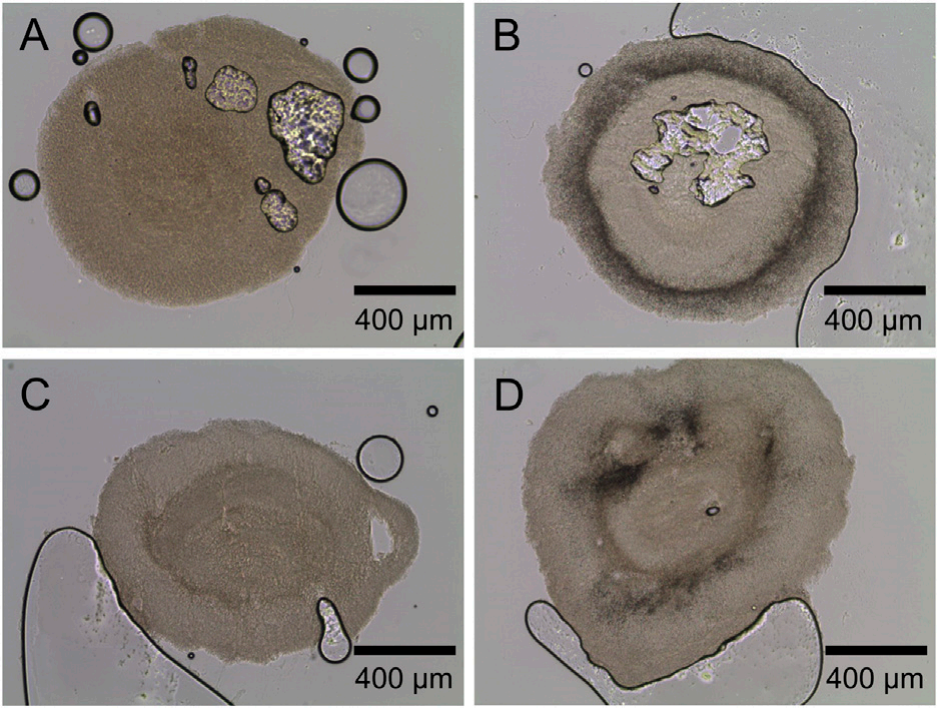
Exemplary microscopic images of sliced pellets from **(A)** unsupplemented control culture. **(B)** MPEC supplemented with glass microparticles (10 g·L^−1^, x_50_ = 7.9 µm). **(C)** Soy lecithin-supplemented culture (SL-IP, 7.5 g·L^−1^). **(D)** MPEC supplemented with glass microparticles (10 g·L^−1^, x_50_ = 7.9 µm) combined with soy lecithin addition (SL-IP, 7.5 g·L^−1^).

First, a screening of varying lecithin species in varying concentrations revealed soy lecithin (EMULPUR™ IP), in the following referred to as SL-IP, in a concentration of 7.5 g·L^−1^ as the best choice for *L. aerocolonigenes* cultivation regarding an optimized rebeccamycin concentration (data not shown). Subsequently, SL-IP was used for further investigations regarding the combination of SL-IP and 10 g·L^−1^ glass microparticles (x_50_ = 7.9 µm). Figure 9 shows the CDW and rebeccamycin concentration and the averaged biomass-related product yield coefficient (AYC) of 10-day cultivation of an unsupplemented control culture, MPEC approach, soy lecithin-supplemented culture, and MPEC combined with soy lecithin addition. Either the supplementation of glass microparticles or lecithin leads to a significant increase in CDW. Moreover, the combination of both leads to an almost threefold increase in CDW. It must be mentioned that the depicted CDWs are the CDWs excluding the mass of microparticles. Accordingly, the rebeccamycin concentration increases slightly due to isolated supplementation of each additive but increases more than fourfold due to combined supplementation and reaches a maximum concentration of 213 mg·L^−1^ after 10 days of cultivation. Considering the AYC, due to an enhanced value for the combined supplementation, it can be concluded that the enhanced rebeccamycin production is not only the result of a higher biomass concentration in the culture broth but also of higher productivity of the cells themselves, e.g., by higher porosity of the biomass (Schrinner et al., 2021) and as a result of an overall reduced AESD of the pellets (data not shown). Schrinner et al. (2021), for example, postulated lecithin to be an additional carbon source resulting in enhanced biomass growth. However, further beneficial effects by combination with glass microparticles cannot be excluded and must be investigated in future studies. Overall, the rebeccamycin titer of 213 mg·L^−1^ obtained here is lower than the one obtained by Schrinner et al. (2021) with glass bead macroparticles and lecithin (388 mg·L^−1^), but in comparison to the previous literature, it is quite high (Nettleton et al., 2019; Schrader et al., 2019; Schrinner et al., 2020). To understand the effect of lecithin supplementation, cross-sections of pellets originating from the four experimental approaches of Figure 9 were investigated. In comparison to the control (Figure 10A), the lecithin supplementation (Figure 10C) led to a more homogeneous hyphae distribution over the entire pellet without areas of lower density (lysed mycelium) in the pellet center. Moreover, both approaches with glass microparticles show differences in the inner structure. Although the cross-sectional slice of Figure 10B (MPEC) shows an incorporation pattern of glass microparticles (darker ring area near the edge) similar to common patterns in Figure 5; the approach of MPEC and additional lecithin supplementation (Figure 10D) lead to a more heterogeneous distribution of the incorporated microparticles with local accumulations.

From the product titers in combination with the cross-sectional images, it can be concluded that lecithin tremendously affects the inner hyphal structure of the pellets, especially when glass microparticles are additionally involved. Thus, it can be assumed that the emulsifier lecithin influences the surface properties of the glass microparticles and effects, e.g., their hydrophobicity. Subsequently, the interaction with hyphae and the microparticle incorporation behavior may be manipulated. Moreover, as rebeccamycin is a strictly hydrophobic substance, it can be hypothesized that lecithin, an emulsifier, may promote rebeccamycin to dissolve into the culture broth, inducing feedback regulatory effects to enhance intracellular product formation. Moreover, the application of lecithin probably results in further effects on culture broth properties. For instance, the oxygen transfer rate or the surface tension is likely to be influenced by supplementation, which again impacts the cultivation and the product formation.

### 3.5 Effect of MPEC on pellet density

MPEC is generally associated with the effect of increasing the pellets’ porosity due to the incorporation of microparticles between hyphae. Thus, investigations were conducted to verify such observations for *L. aerocolonigenes* with varying glass microparticle concentrations (2.5, 5, 7.5, 10, and 12 g·L^−1^). Gravimetric measurements of MPEC pellets (Table 1) show that the 7.5 g·L^−1^ approach is an exception, significantly reducing pellet densities up to almost 40% of the initial pellet density. Due to the vague method, the determined values should be rather seen as a rough estimation than as reliable absolute values, as, for instance, incorporated microparticles were included during gravimetric determination. Furthermore, only a specific size class within all pellet sizes is considered, and the method is based on the assumption that pellets for wet volume determination are spherical objects, which often do not meet reality. However, a clear trend emerges from the data.

**TABLE 1:** Overview of the pellet density, the maximum oxygen gradient ( $\frac{dSatO_{2}}{dr}$ )_max_, the active oxygen supplied pellet layer (∆r_activ_), and the rebeccamycin concentration after 10 days of cultivation corresponding to the glass microparticle concentration of the culture.

Glass microparticle concentration (g L^−1^)	Pellet density (kg m^−3^)	( $\frac{dSatO_{2}}{dr}$ )_max_ (% µm^−1^)	∆r_activ_ (µm)	Rebeccamycin concentration (mg L^−1^)
0	106.49	0.53 ± 0.03	300 ± 16	0.3 ± 0.1
2.5	73.92	0.38 ± 0.04	393 ± 43	7.1 ± 1.4
5	46.93	0.41 ± 0.03	300 ± 28	10.7 ± 2.5
7.5	111.29	0.53 ± 0.02	250 ± 8	15.1 ± 3.5
10	56.52	0.45 ± 0.06	287 ± 28	22.3 ± 5.6
12	46.70	0.60 ± 0.08	237 ± 34	46.1 ± 10.0

**FIGURE 11:**
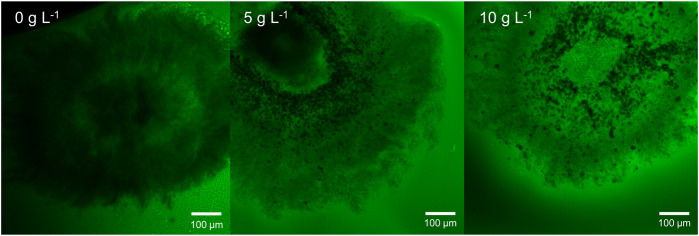
Exemplary microscopic images of sliced pellets from an unsupplemented control culture and MPEC supplemented with glass microparticles (x_50 = 7.9 µm) at concentrations of 5 and 10 g L^−1^. Staining was performed with dextran fluorescein (10,000 Da). Green areas represent low hyphal density.

For validation of the described gravimetric density measurements, CLSM was applied to detect the pellet densities visually. Fluorescein-labeled dextran is a high-molecular inert polymer incapable of entering cells. It can be used to assess the diffusion conditions representing the porosity within pellets as an increased fluorescence signal represents more accumulated molecules in the intrahyphal area, which correlates with higher porosity. The resulting pellet images of the unsupplemented control and the culture supplemented with 5 and 10 g·L^−1^ glass microparticles are shown exemplarily in Figure 11, representing a rather qualitative than quantitative assessment. As the threshold signal was similar for every slice, comparability is given.

Several trends can be derived from Figure 11. Although the pellet slice of the unsupplemented control (without microparticle addition) appears dense and only an inner ring of low density (lighter color) can be located, the incorporated glass microparticles (5 and 10 g·L^−1^) can be seen as black accumulations encircling the pellet center. Among increasing microparticle content, a general signal increase in fluorescein can clearly be seen visually in the presented example and for the further microparticle concentrations (2.5, 7.5, and 12 g·L^−1^) (data not shown) by overall lighter greenish pellet structure. Moreover, areas with incorporated microparticles in the pellet appear in lighter green compared to the outer regions. In the original method, the resulting CLSM pictures were converted to eight-bit greyscale images, and subsequently, image analysis was performed to classify pixels to be either pore or hyphae. Thus, radial density profiles were performed (Hille et al., 2005). This advantageous procedure is not possible for the investigated pellets. First, the hyphae in actinomycetes are far more filigree than fungal species (Bush et al., 1987; Schrinner et al., 2020) to be resolved by CLSM as defined structures. Second, the incorporated microparticles emerge clearly in the picture and disturb radial density profiling. All in all, by summarizing both the gravimetric and the CLSM approach, there is strong evidence that the hypothesis of MPEC resulting in lowering the hyphal density can be confirmed for *L. aerocolonigenes* cultivated with glass microparticles.

### 3.6 Oxygen supply in MPEC pellets

**FIGURE 12:**
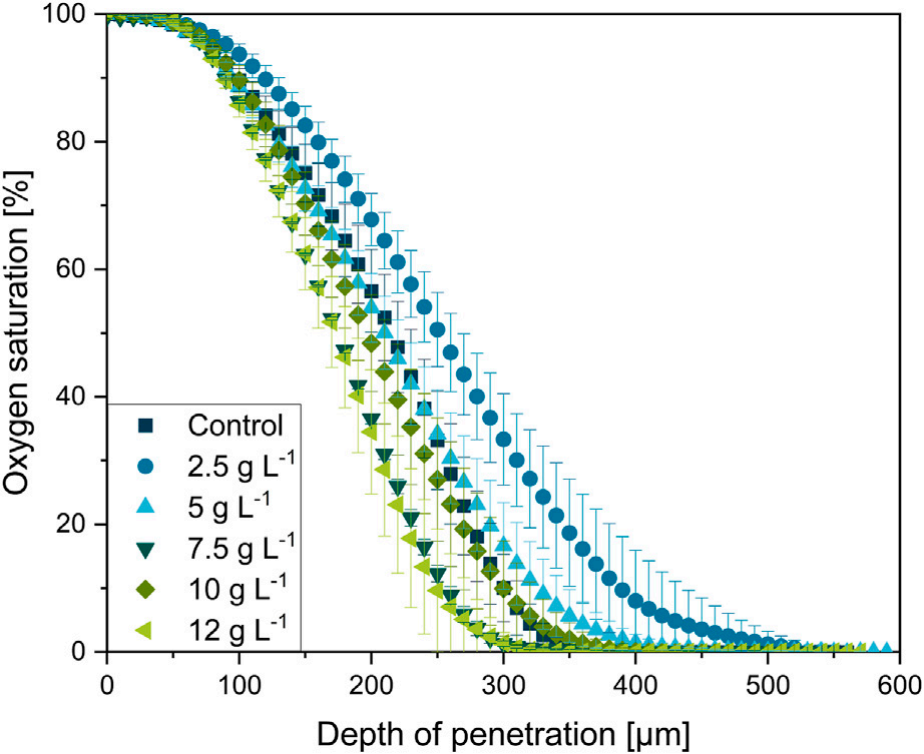
Oxygen microprofiling experimentally acquired with the experimental setup depicted in Figure 1. Oxygen saturation as a function of in dependance on the relative penetration depth into the *L. aerocolonigenes* pellet for varying microparticle concentrations in the culture broth. Pellets were investigated after 64 h of cultivation and were selected within a similar size range.

The results in Section 3.5 verify the hypothesis of pellet porosities being increased by microparticle supplementation. Originating from these structural changes, it can be assumed that the mass transfer of oxygen and nutrients into the pellet may be enhanced. To validate this common hypothesis, oxygen microprofiling measurements were conducted in pellets of *L. aerocolonigenes* and, thus, executed in filamentous bacteria for the first time. The same pellets investigated in Section 3.5 (CLSM measurements) were used for oxygen profiling. An unsupplemented control approach and cultivation with glass microparticle (x_50_ = 7.9 µm) supplementation (2.5, 5, 7.5, 10, and 12 g·L^−1^) were conducted to evaluate the impact of incorporated microparticles on local oxygen concentration inside pellets. A minimum of three or four pellets of similar pellet size were investigated for each microparticle concentration, and at least three profiling cycles per pellet were conducted to assure certain statistical reliability. Profiles were normalized to 100% oxygen saturation (oxygen concentration in bulk phase) and were decoupled from their radial coordinate to correct inaccuracies of pellet surface determination. In Figure 12, the resulting oxygen profiles are presented.

In physical proximity to the pellet bulk interface (0 µm depth of penetration, 100% oxygen saturation), the oxygen concentration initially remains steady for all oxygen profiles before the major decrease starts in a layer of 100 µm pellet depth. The steep decrease terminates at about 200–400 µm in the oxygen-limited inner pellet area. Thereby, microparticle-dependent variances in the curve progressions can be observed. Emanating from the profile of the control cultivation, the profile of the 2.5 g·L^−1^ cultivation is significantly flatted, which equivalent to a shallow oxygen gradient deep into the pellet center. By considering the working hypothesis that supplementation of microparticles leads to higher pellet porosity and, thus, a better and deeper mass transfer of oxygen, the flatted profile coincides well with expectations. However, a further increase in microparticle concentration leads to a trend in the opposite direction. With increasing microparticle concentration the oxygen profiles gain steepness. As the pellets used for oxygen profiling originate from the same cultures as for the density elucidation (Section 3.5), the 7.5 g·L^−1^ approach is consistently an exception, probably due to deviations during cultivation, as the cultures are diverse biological approaches. Thus, the oxygen consumption in the outer pellet layer increases, which contradicts the trend of pellet densities.

The same trend can also be seen in the maximum oxygen saturation gradient (dSatO_2_/dr)_max_, showing the 12 g·L^−1^ supplementation to be the approach with the highest oxygen consumption over the radial pellet coordinate with 0.60 ± 0.08 µm^−1^ (see Table 1). Furthermore, Table 1 shows that the oxygen-consuming pellet layer ∆r_active_ (the layer between 98% and 1% oxygen saturation) tends to decrease with microparticle concentration according to rather steeper oxygen gradients. Still, rebeccamycin concentration increases with every increase in microparticle concentration (see Table 1). Hence, the faster decrease in oxygen supply seems not to be disadvantageous for the overall production performance.

In addition to the assumption that the outer active pellet layer supplied with oxygen becomes thick, the enhanced hyphal activity and viability (shown in Section 3.3 and Driouch et al. (2010a)) must be considered. Enhanced viability and activity entail a higher oxygen consumption in the specific pellet region. Nonetheless, oxygen microprofiling in *L. aerocolonigenes* was also conducted with pellets originating from glass bead macroparticle cultivation, as it is reported in previous studies (Schrader et al., 2019; Schrinner et al., 2020; Schrinner et al., 2021) and in which an increase in rebeccamycin production and higher viability were reported, too. However, no clear trend could be detected within this experimental approach (data not shown). Therefore, it can be concluded that the affection of oxygen profiles is exclusive to MPEC.

In summary, the measured oxygen profiles in pellets from MPEC do not only reflect enhanced oxygen transport into the pellets due to decreased hyphal density. In fact, the enhanced oxygen demand of more active pellet layers is also represented, which correlates with the pure presence of the microparticles and, thus, the shown decreased hyphal density. Thus, it is not necessarily the better oxygen supply through a denser active pellet layer due to the looser structure that is beneficial for the product formation, but also the interplay of all the mentioned factors. Moreover, a recent review by Laible et al. (2021) sheds light on further possible aspects potentially playing crucial roles in MPEC. For future studies, it is important to investigate, in detail, how MPEC and the change in microparticles (e.g., dissolution and the release of ions) influence the physicochemical properties of the cultivation broth and, thus, the change in cellular morphology and the performance of product formation.

## 4 Conclusion

This work introduces glass microparticles with a median size of 7.9 µm as a novel system for a more precise cultivation setup for MPEC. Glass microparticles induced superior rebeccamycin synthesis in *L. aerocolonigenes* than control cultures and a talc-supplemented approach. Thereby, by significantly reducing pellet sizes, a broad incorporation of microparticles into the hyphal pellet structure and significantly increased rebeccamycin titers were observed. Monitoring the side effects of MPEC, the presence of microparticles preserves the inner pellet from early decay of cellular integrity and cell death, as the overall viability remains on a higher level compared to unsupplemented control cultivation. Furthermore, pellet porosity tends to increase due to microparticle supplementation confirming early hypotheses. However, contradictory to the previously postulated theories, the increase in oxygen consumption in more active cells leads to a steeper oxygen gradient with increasing particle supplementation. Moreover, soy lecithin supplementation was investigated in this study in addition to MPEC. Compared to an unsupplemented control culture, a more than four-fold increased rebeccamycin titer of 213 mg·L^−1^ shows the highest titer published so far for MPEC of *L. aerocolonigenes*. Especially in regard to a potential scale-up, MPEC seems to be a promising way to enhance the cultivation performance of filamentous microorganisms in bioreactors. The MPEC implementation appears to be rather uncomplicated in comparison to larger *macro*particles (despite the fact that macroparticles seem to be slightly beneficial for productivity in shaking flasks). However, a scale-up with glass microparticles and its characteristics will be investigated in upcoming studies. So far, a few articles can be found addressing MPEC being applied in reactors with a volume of up to 30 L (Gonciarz et al., 2016; Kowalska et al., 2017; Yatmaz et al., 2020; Gürler et al., 2021). A further step into an even larger scaled stirred tank bioreactor may entail challenges (e.g., abrasion and downstream processing) and has to be intensively investigated in the future.

All in all, this work provides a foundation for further studies focusing on the underlying effects of microparticles during MPEC. It can be theorized that several potential physicochemical effects could play crucial roles in the interaction of microparticles with hyphae. Thus, the glass microparticles introduced here may operate as a precisely tunable backbone for detailed research on this topic, making MPEC an even more powerful tool for tailor-made adjustments in filamentous cultivations.

## Data Availability

The raw data supporting the conclusion of this article will be made available by the authors without undue reservation.

## Nomenclature

AYC: Averaged biomass-related product yield coefficient (mg_Rebeccamycin_ g_CDW_^-1^)
CDW: Cell dry weight concentration (g L^−1^)
CLSM: Confocal laser scanning microscopy
MM: Micromanipulator
MPEC: Microparticle-enhanced cultivation
MW: Molecular weight (Da)
PI: Propidium iodide
RT: Room temperature
SD: Standard deviation
SL-IP: Soy lecithin
x: particle size (µm)
